# Importance of retrograde axonal transport in mitochondrial health and distribution

**DOI:** 10.1038/s41420-021-00502-3

**Published:** 2021-05-15

**Authors:** Christy Hung

**Affiliations:** UCL Great Ormond Street Institute of Child Health, London, UK

**Keywords:** Mitochondria, Cellular neuroscience

Mitochondria acts like powerhouses of the cells, providing cellular energy in the form of ATP molecules to support almost all essential cellular processes, from the maintenance of intracellular calcium homoeostasis to the regulation of cell death and survival^1^. Neurons, which consume 20% of the body’s basal oxygen, are highly dependent on mitochondrial respiration to fuel their critical function^2^. Healthy mitochondria are indispensable at synapses for the maintenance of functional neural circuits as local ATP levels are required for the release and recycling of synaptic vesicles on both the pre- and postsynaptic sides. Therefore, robust long distance transport machinery is required to ensure continuous supply of healthy mitochondria to the regions of high energy demands and to clear aged and malfunctioning mitochondria from the axon terminals to prevent the build-up of toxic reactive oxygen species (ROS). However, the extreme length of axonal processes and arborization present a tremendous transport challenge for the neurons to maintain a healthy and functioning pool of mitochondria throughout the axons^1^.

Emerging evidence suggests that mitochondrial transport impairments is a common feature across many neurodegenerative diseases^3^, including amyotrophic lateral sclerosis (ALS). ALS is a progressive neurological disease affecting primarily the motor neurons, which have exceptionally high energy demands when compared to other neurons. This high dependence on the continuous supply of ATPs to power their synaptic activities makes motor neurons crucially dependent on the trafficking of mitochondria to the synapses and render them particularly vulnerable to disturbances in energy metabolism. In fact, mitochondrial transport defects have been observed as early as at embryonic stage in mouse models of ALS, suggesting that mitochondrial transport impairments are likely a cause rather than the consequence of the disease^4^.

Restoring axonal transport has been proposed as an important therapeutic target against ALS. But how is axonal mitochondrial transport regulated? Active transport of mitochondria in neurons is driven by molecular motor proteins. Kinesin-1 and cytoplasmic dynein motors mediate the anterograde and retrograde transport of mitochondria respectively^5^. Mitochondria attached to motor proteins via motor adaptor proteins. Milton (also known as TRAK1/2) is an adaptor protein associated with the heavy chain of kinesin-1 and interacts with Miro (also known as RhoT1/2), a protein anchored to the mitochondrial outer membrane^6^. Together, milton/miro/kinesin-heavy chain constitute a complex for the anterograde transport of mitochondria. While we are beginning to understand the mechanics of mitochondrial anterograde transport, the mechanisms and function of mitochondrial retrograde transport remains largely unexplored.

Recent work by Mandal et al. significantly extended our understanding on the importance of retrograde mitochondrial transport in neurons and shed new lights on the consequence of disrupting this process on sensory and motor circuits in vivo^7^. The authors first visualise the distribution of fluorescently tagged mitochondria (mito-TagRFP) in vivo in zebrafish posterior lateral line (pLL) sensory neurons^8^ carrying loss-of-function mutation in *actr10* (*actr10*^*nl15*^), which has previously been shown by Drerup et al. to inhibit retrograde mitochondrial transport specifically without affecting the anterograde movement^9^. The authors observed a massive accumulation of mitochondria at the axon terminals accompanied with a significant reduction of mitochondrial load in the cell bodies of the *actr10*^*nl15*^ mutant neurons. They further verified their findings by (i) characterising the mitochondrial distribution in pLL axons of *p150a/b* double mutants, which shifted the distribution of mitochondria in a manner similar to the loss of Actr10; and (ii) using siRNA-mediated knockdown of *actr10* in cultured rat hippocampal neurons. Together, these results suggested that inhibition of mitochondrial retrograde transport results in an imbalanced mitochondrial load and indicated that the *actr10*^*nl15*^ zebrafish line is a useful model to investigate the downstream effects of disrupting retrograde mitochondrial transport in vivo.

Mandal et al. next evaluated whether inhibiting retrograde transport would impact mitochondrial health. The authors used various fluorescence probes to measure ROS production and the matrix potential of the mitochondria in the *actr10*^*nl15*^ pLL sensory neurons. They showed that those mitochondria retained in the axon terminals have (i) elevated measure of chronic ROS; (ii) lowered acute ROS production; and (iii) reduced matrix potential. On the contrary, using the same fluorescence indicators, they found that those mitochondria in the cell bodies remained functional intact as reflected by normal ATP production and have lowered chronic ROS exposure, which is likely an indication of bulk mitochondrial biogenesis taking place in cell bodies. These analyses demonstrated that disrupting retrograde mitochondrial motility leads to the loss of cell body mitochondria and accumulation of damaged organelles in axon terminals, further highlighting the importance of retrograde axonal transport in the distribution of a healthy mitochondrial population throughout the neurons.

While there is no question that axonal transport of mitochondria is essential for maintaining neuronal health as mitochondrial transport impairments is emerging as a common feature across many neurodegenerative diseases^3^, the question of why mitochondria move bidirectionally in the axons is less clear. It is generally assumed that anterograde transport is responsible for supplying healthy mitochondria to the axon terminals from the site of biogenesis near the cell body and the main purpose of retrograde mitochondrial transport is to remove damaged organelle from the axons for degradation. Surprisingly, by imaging the localisation of mitochondria labelled with photoconvertible proteins over the scale of hours and days, Mandal et al. showed that mitochondrial turnover occurs consistently within 24 h at synapses in healthy neurons. Importantly, they found that not all mitochondria leaving the axon terminals are degraded but rather persisted over several days for redistribution throughout the axons.

**Fig. 1:**
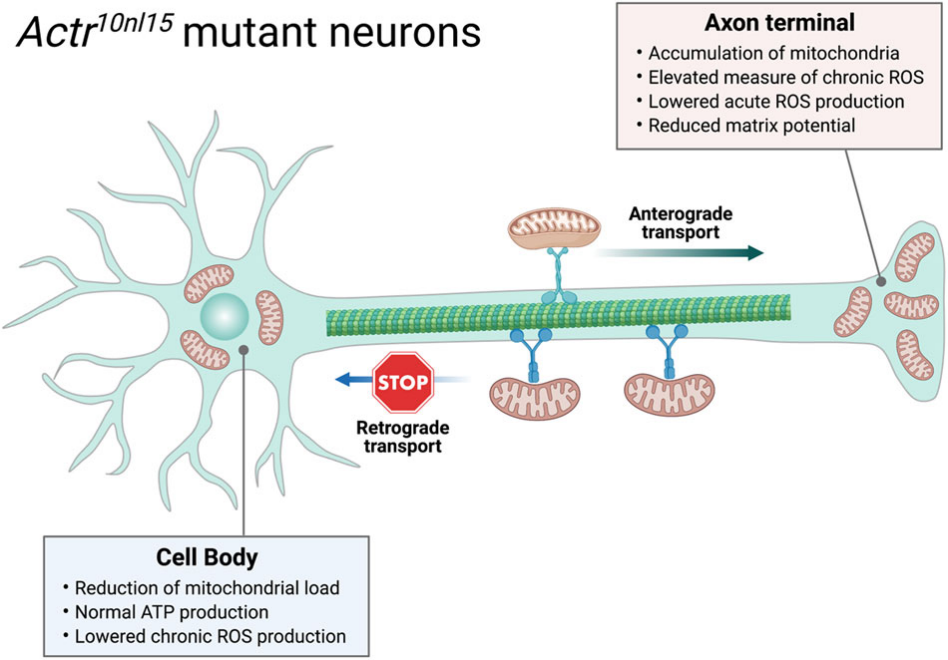
Inhibition of mitochondrial retrograde transport results in an imbalanced mitochondrial load. (Created with BioRender.com.).

These exciting new findings by Mandal et al. offer fundamental insight into the molecular mechanisms underlying the bidirectional transport of mitochondria in vivo. The authors provide compelling evidence demonstrating that retrograde mitochondrial transport serves not only just to prevent the accumulation of aged organelles in distal axons but also plays critical roles in maintaining the uniformed distribution of a healthy mitochondria pool throughout the axons (Fig. 1). These new findings by Mandal et al. open up new avenues to explore whether signalling effectors regulating retrograde transport might be novel targets for restoring mitochondrial health and distribution in ALS and other neurodegenerative conditions involving mitochondria trafficking defects.
